# The dissociation between command following and communication in disorders of consciousness: an fMRI study in healthy subjects

**DOI:** 10.3389/fnhum.2015.00493

**Published:** 2015-09-15

**Authors:** Natalie R. Osborne, Adrian M. Owen, Davinia Fernández-Espejo

**Affiliations:** ^1^The Brain and Mind Institute, University of Western OntarioLondon, ON, Canada; ^2^Department of Psychology, University of Western OntarioLondon, ON, Canada

**Keywords:** functional magnetic resonance imaging (fMRI), disorders of consciousness, command following, communication, motor execution, motor imagery

## Abstract

Neuroimaging studies have identified a subgroup of patients with a Disorder of Consciousness (DOC) who, while being behaviorally non-responsive, are nevertheless able to follow commands by modulating their brain activity in motor imagery (MI) tasks. These techniques have even allowed for binary communication in a small number of DOC patients. However, the majority of patients who can follow commands are unable to use their responses to communicate. A similar dissociation between present command following (CF) and absent communication abilities has been reported in overt behavioral assessments. However, the neural correlates of this dissociation in both overt and covert modalities are unknown. Here, we used functional magnetic resonance imaging (fMRI) to explore the neural mechanisms underlying CF and selection of responses for binary communication using either executed or imagined movements. Fifteen healthy participants executed or imagined two different types of arm movements that were either pre-determined by the experimenters (CF) or decided by them (action selection, AS). Action selection involved greater activity in high-level associative areas in frontal and parietal regions than CF. Additionally, motor execution (ME), as compared to MI, activated contralateral motor cortex, while the opposite contrast revealed activation in the ipsilateral sensorimotor cortex and the left inferior frontal gyrus. Importantly, there was no interaction between the task (CF/AS) and modality (MI/ME). Our results suggest that the neural processes involved in following a motor command or selecting between two motor actions are not dependent on how the response is expressed (via ME/MI). They also suggest a potential neural basis for the distinction in cognitive abilities seen in DOC patients.

## Introduction

In recent years, advances in neuroimaging techniques have made it possible to detect signs of covert cognition in patients with a clinical diagnosis of vegetative state (VS; Fernández-Espejo and Owen, 2013). VS patients do not show purposeful overt behavior and thus are considered to be entirely unaware of themselves and their environment (Jennett and Plum, 1972). However, it is estimated that around 20% of them may be able to follow commands by willfully modulating their brain activity in mental imagery tasks (Monti et al., 2010; Cruse et al., 2011). Such tasks typically involve instructing the patient to imagine a motor action (e.g., swinging their arm to hit a tennis ball; Owen et al., 2006), while their neural responses are recorded with functional magnetic resonance imaging (fMRI) or electroencephalography (EEG; for a review of these studies, see Fernández-Espejo and Owen, 2013).

To date, 34 VS and other non-responsive patients with a disorder of consciousness (DOC) have demonstrated covert command following (CF) in motor imagery (MI) tasks with EEG (Cruse et al., 2011, 2012a; Gibson et al., 2014; Horki et al., 2014; Coyle et al., 2015), or fMRI (Owen et al., 2006; Monti et al., 2010; Bardin et al., 2011; Fernández-Espejo and Owen, 2013; Forgacs et al., 2014; Gibson et al., 2014). Subsequent studies have used selective visual or auditory attention (Schnakers et al., 2008; Lulé et al., 2013; Naci and Owen, 2013; Monti et al., 2014; Pan et al., 2014), as well as attempted movements (Bekinschtein et al., 2011; Cruse et al., 2012b; Horki et al., 2014) to reveal covert awareness in 34 more patients. The reliability of fMRI for detecting when participants are imagining a motor command, or engaged in other mental imagery tasks (e.g., imagining walking around their house) has allowed some of the approaches above to be successfully used as communication tools, by pairing each pattern of activity with “yes” and “no” responses (Fernández-Espejo and Owen, 2013). However, the majority of patients who successfully follow commands are unable to perform communication tasks (Owen, 2011). Indeed, to date only three DOC patients have been able to successfully communicate accurate answers to yes/no questions in the scanner (Monti et al., 2010; Fernández-Espejo and Owen, 2013; Naci and Owen, 2013), while a fourth exhibited communication capabilities but failed to produce correct answers (Bardin et al., 2011).

Command following and communication are well-established signs of consciousness (Giacino et al., 2004) and as such, are systematically explored in standard bedside diagnostic assessments. The Coma Recovery Scale-Revised (CRS-R; Giacino et al., 2004), an internationally accepted behavioral diagnostic tool for DOCs, considers reliable behavioral responses to commands one of the key diagnostic criteria to reclassify a patient as being in a minimally conscious state (MCS; Giacino et al., 2002). Moreover, when present, reliable CF guarantees further assessment of communication capabilities. Importantly, only when communication becomes functional (i.e., the patient is able to give accurate answers) is the patient considered to be emerging from the MCS (Giacino et al., 2002). MCS patients are known to be clinically heterogeneous, but very few works have systematically studied the occurrence of behavioral CF or communication. A recent report including a cohort of 52 MCS patients identified CF in 33%, and non-functional communication in 19% of them. Importantly, only 17% of chronic patients who were assessed more than 1 year after the initial injury showed CF abilities, and none were able to communicate (Estraneo et al., 2014).

The ability to communicate correct answers depends on preservation of a number of high-order cognitive processes, such as autobiographical memory, semantic representations, mental orientation, etc. However, when accuracy is not taken into account (non-functional communication), providing responses to binary questions ultimately requires the ability to select between two alternative behaviors, representing “yes”/“no”. The specific mechanisms underlying the differences between the ability to respond to a command, and the ability to select between two potential responses to answer a binary question (henceforth referred here as “CF” and “action selection, AS” respectively) have not been explored. Furthermore, the relationship between such differences and the type of behavior (mental or behavioral) used to provide the responses is entirely unknown.

In order to investigate these questions, we designed an fMRI paradigm where healthy participants were asked to move their right hand (motor execution, ME) or imagine moving their right hand (MI) in response to auditory cues. Such cues instructed them to either voluntarily select an action between two possible alternatives, or perform the one that was dictated to them.

## Materials and Methods

### Participants

Fifteen right-handed healthy volunteers (ages 19–29, average 24 years; eight females) with no history of neurological or psychiatric disease participated in the study. All volunteers gave written informed consent and were compensated for their participation in the experiment. The Health Sciences Research Ethics Board of the University of Western Ontario provided ethical approval for the study.

### fMRI Paradigm

Participants lay supine with their right arm bent at an approximately 90° angle so that their forearm rested across their torso. Because movements of the shoulder and upper arm may induce artifacts in the participant’s data (Rossit et al., 2013), a strap around the participant’s chest was used to minimize upper arm and shoulder movements, while allowing for full rotation at the elbow.

**Figure 1:**
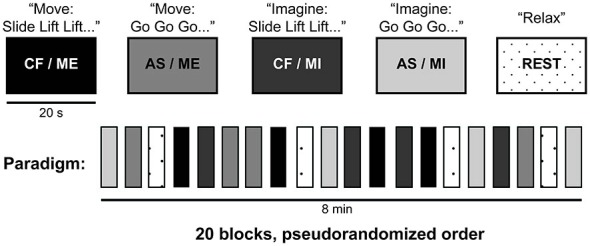
**fMRI paradigm**. This study combined two different arm movements (slide and lift) to create various six-movement long sequences (e.g., “slide lift lift slide lift slide”). Subjects either executed (ME) or imagined (MI) movement sequences that were either pre-determined by experimenters (CF) or chosen by them (AS), in a 2 × 2 within subjects factorial design. Onsets in CF blocks were cued with the words “slide”, or “lift”, while “go” was used to cue onsets in AS blocks. Each block lasted 20 s and consisted of six moves. During rest blocks (dotted), subjects lay still in the scanner. There were four blocks of each condition, which were presented in a pseudorandom order for a total of 20 blocks (in an additional condition not reported here, the experimenter passively moved subjects’ arms). For the CF blocks, subjects were randomly assigned four different movement sequences from a collection of 48 unique, pre-determined movement sequences created for this experiment. CF, command following; AS, action selection; ME, motor execution; MI, motor imagery.

Figure 1 described the fMRI paradigm used in this experiment. While in the MRI scanner, participants were instructed to either execute or imagine a series of movements involving their right forearm. We used two different arm movements: a “slide”, which involved sliding the forearm forward and back; and a “lift”, which involved lifting and lowering the forearm. Each sequence involved six movements (combining “slides” and “lifts”). Imagery and execution blocks were 20 s long, and were alternated with periods of rest for a total of 8 min. The beginning of each block was cued with the word “move”, “imagine” or “relax”. Participants also completed blocks where they were instructed to relax while a researcher moved their arm (data not reported here). Within each block (imagery or execution), participants either received a pre-determined sequence (i.e., CF) or were asked to create one by individually choosing one out of the two possible movements at a time (AS), in a 2 × 2 within-subjects factorial design. During the blocks with pre-determined sequences, each individual action was cued with the word “slide” or “lift”. Each participant was randomly assigned four out of a possible 48 unique movement sequences, all four of which were presented pseudorandomly over each experimenter-cued condition. For those where the subject had to create their own sequence, each action was cued with the word “go”. There were four blocks of each condition, which were presented in a pseudorandom order for a total of 24 blocks. All participants completed two runs of this task. An infrared MR-compatible camera (MRC Systems GmbH), placed above the participant’s head, was used to record participants’ actions for each run. The recordings were monitored online to confirm that all participants performed all runs with no errors (i.e., they moved their hand to command during ME trials, and remained still during MI trials). In addition to the video monitoring, participants were asked afterwards about their execution of the task. All participants reported performing the imagery task correctly.

### Image Acquisition

Data was acquired in a 3T Siemens scanner (Magnetom Prisma, Siemens, Germany), with a Siemens 32-channel head-coil, at the Centre for Functional and Metabolic Mapping (CFMM) at Robarts Research Institute. Audio instructions and task cues were presented using Matlab® R2011a on a MacBook Pro laptop (OSX 10.6.8) and an MRI-compatible high-quality digital sound system via noise-attenuated headphones (Sensimetrics, S14).

The fMRI protocol included two sessions of 240 volumes each, using echo-planar images (36 axial slices, *TR* = 2000 ms, *TE* = 30 ms, matrix size = 70 × 70, slice thickness = 3 mm, in-plane resolution = 3 × 3 mm, flip angle = 78°). A high-resolution T1-weighted MPRAGE structural image (*TR* = 2300 ms, *TE* = 2.32 ms, *IT* = 900, matrix size = 256 × 256, voxel size 1 × 1 × 1 mm, flip angle = 8°) was also acquired.

### fMRI Data Analysis

We performed Independent Component Analysis using the FSL MELODIC tool,^1^ in order to remove motion artifacts (Friston et al., 1996; McKeown and Sejnowski, 1998; Beckmann and Smith, 2004). One of the authors (N.R.O.) visually inspected all the components and identified those that corresponded to head-motion artifacts and were correlated with the execution blocks. An average of 5 ± 2.6 artifactual components were identified per subject and run. Finally, we removed the identified components from the fMRI data. The de-noised data was then pre-processed and analyzed with SPM8.^2^ After manually AC-PC reorienting the data, the following spatial pre-processing steps were performed: realignment, co-registration of the structural and functional data, spatial normalization to Montreal Neurological Institute (MNI) space, and smoothing with an 8-mm FWHM Gaussian kernel. High-pass filtering with a cut-off period of 128 s was used to remove linear drift. A single subject fixed-effect two-by-two factorial analysis was performed for each subject at the whole-brain level. Factor 1 was defined as “Task” with two levels (motor imagery/motor execution) and Factor 2 was defined as “Level of selection”, with two levels (AS/CF). Scans were modeled as belonging to the AS/ME, CF/ME, AS/MI, or CF/MI conditions using the canonical hemodynamic response function (Friston et al., 1995) with the participant’s rest condition used as a baseline. Realignment parameters and passive movement blocks were modeled as effects of non-interest.

While all participants reported completing the MI blocks, the nature of MI precludes any observable or external means of confirmation that they did indeed perform the task. However, previous MI studies have demonstrated that activity in the supplementary motor area (SMA) can be used as neural evidence for MI (Owen, 2011). We examined individuals’ whole brain activity during MI conditions compared to rest in order to confirm their completion of the task, and to avoid biases in the analysis from including participants who may not have performed it. 13 out of 15 participants showed significant activity in the SMA (cluster level uncorrected *p* < 0.001). The remaining two participants were removed from subsequent analyses. Therefore, 13 participants were included in the group analyses, which consisted of one-sample t-tests for each contrast of interest. The statistical threshold was set at a family wise error (FWE) corrected *p* < 0.05 at the cluster-level. Two additional contrasts, individually comparing ME and MI conditions to rest, were also included to confirm that the task elicited a similar pattern of activation as previously reported paradigms (Owen et al., 2006, 2007; Formaggio et al., 2013; Machado et al., 2013; Fernández-Espejo et al., 2014). The FSL Harvard-Oxford Cortical and Subcortical Structural Atlases (see Acknowledgments) were used for anatomical identification.

## Results

### Motor Imagery vs. Motor Execution

**Figure 2:**
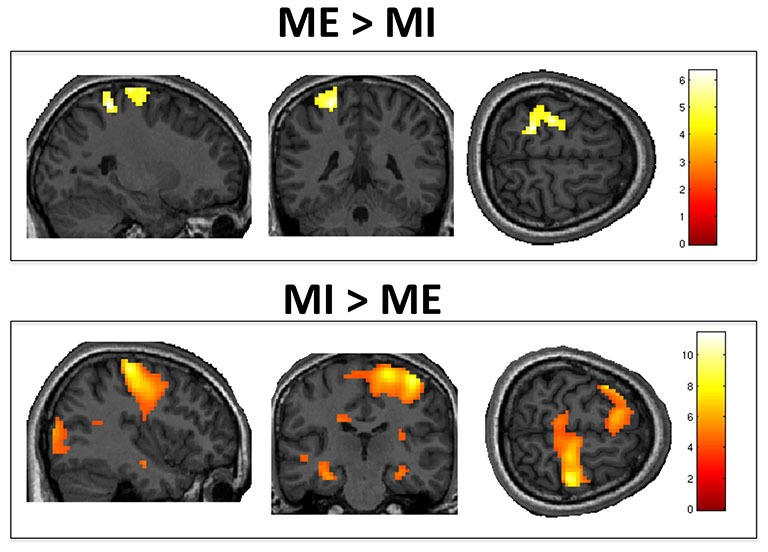
**Motor execution vs. motor imagery**. Group level analysis showed greater activity in contralateral M1/S1 when motor execution was compared to motor imagery (top panel). Conversely, motor imagery, as compared to motor execution, activated the ipsilateral M1, S1 and left inferior frontal gyrus (bottom panel). Results are thresholded at family wise error (FWE)-corrected *p* < 0.05 for cluster level activation and overlaid on an anatomical T1-weighted image.

The positive effect of *task* (i.e., ME vs. MI) revealed a significant cluster of activation in the left sensorimotor area, as shown in Figure 2. This included M1, the primary somatosensory cortex (S1), and the superior parietal lobule. The negative effect of *task* (i.e., MI vs. ME) revealed significant activity in the right S1 and M1, left inferior frontal gyrus and right occipital pole (representing the primary and secondary visual cortices). Group activations are shown in Table 1.

**Table 1:** **Motor execution vs. motor imagery**.

Brain structure	Coordinates			Cluster size (*k*)	*T* value	*p* value
	x	y	z
**Positive effect of task (motor execution > motor imagery)**
Superior parietal lobule/postcentral gyrus	−24	−43	61	269	6.35	0.01
**Negative effect of task (motor imagery > motor execution)**
Inferior frontal gyrus	−57	20	22	5731	11.44	<0.001
Postcentral/Precentral gyrus	39	−25	61	1680	9.18	<0.001
Occipital pole	12	−88	28	718	7.17	<0.001

*Results thresholded at FWE-corrected p < 0.05 for cluster level activation. Coordinates are in Montreal Neurological Institute (MNI) space, anatomical structures were identified using the FSL Harvard-Oxford Cortical and Subcortical Structural Atlases*.

### Action Selection vs. Command Following

**Figure 3:**
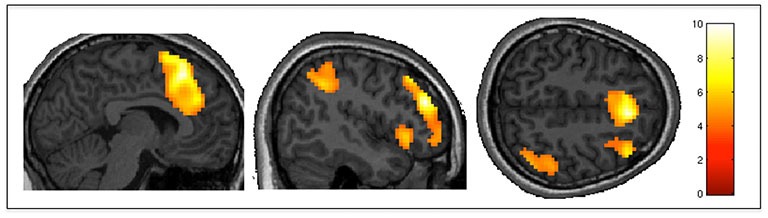
**Action selection vs. command following**. AS (participants selected their movements), as compared to CF (movements were determined by experimenters) elicited greater activity in the middle frontal gyrus, pre-SMA, somatosensory association cortex and insula. Results are thresholded at FWE-corrected *p* < 0.05 for cluster level activation, and overlaid on an anatomical T1-weighted image.

The positive effect of *level of selection* (i.e., conditions where the participant had to choose between two actions vs. those in which the action was determined by the experimenter) revealed significant activity in frontal regions including bilateral frontal poles and middle frontal gyri, as well as the paracingulate gyrus (including pre-SMA). There was also significant activation in the somatosensory association cortex, specifically the right angular gyrus and the left insular cortex. Group activity for this contrast is shown in Figure 3. The inverse contrast (CF vs. AS) showed bilateral activation in the lateral occipital cortex (extrastriate visual area) and primary auditory cortices as well as the precuneus cortex. Group activations are shown in Table 2.

**Table 2:** **Action selection vs. command following**.

Brain structure	Coordinates			Cluster size (*k*)	*T* value	*p* value
	x	y	z
**Positive effect of level of selection (*action selection > command following*)**
Frontal pole, Middle frontal gyrus	48	38	25	2662	9.97	<0.001
Middle frontal gyrus	−36	29	31	113	8.34	0.045
Angular gyrus	45	−49	40	281	6.06	0.001
Frontal pole	−24	50	−14	278	5.29	0.001
**Negative effect of level of selection (*command following > action selection*)**
Middle temporal gyrus, Lateral occipital cortex	48	−58	4	229	11.85	<0.003
Lateral occipital cortex	−48	−70	10	148	8.50	0.02
Superior temporal gyrus	−63	−19	1	347	6.88	<0.001
Heschl’s gyrus	48	−13	1	169	6.17	0.012
Precuneus cortex	−12	−58	13	312	5.36	0.001

*Results thresholded at FWE-corrected p < 0.05 for cluster level activation. Coordinates are in Montreal Neurological Institute (MNI) space, anatomical structures were identified using the FSL Harvard-Oxford Cortical and Subcortical Structural Atlases*.

### Interactions

There were no significant interactions between *task* and *level of selection*. No activity was observed even when thresholds were lowered to an uncorrected *p* < 0.01. To increase the sensitivity of our exploration of this interaction, we ran an additional analysis using a mask including all areas active in the main effect of task and level of selection. The result from this additional analysis confirmed no significant effects, even at uncorrected *p* < 0.01. To further explore the consistency of this (lack of) effect at an individual participant level, we inclusively masked the positive interaction for each individual with their activity from the two main effects. This revealed no significant activity for any participant. Two participants however showed an uncorrected cluster in the left frontal pole, with peak below an uncorrected *p* < 0.001.

**Figure 4:**
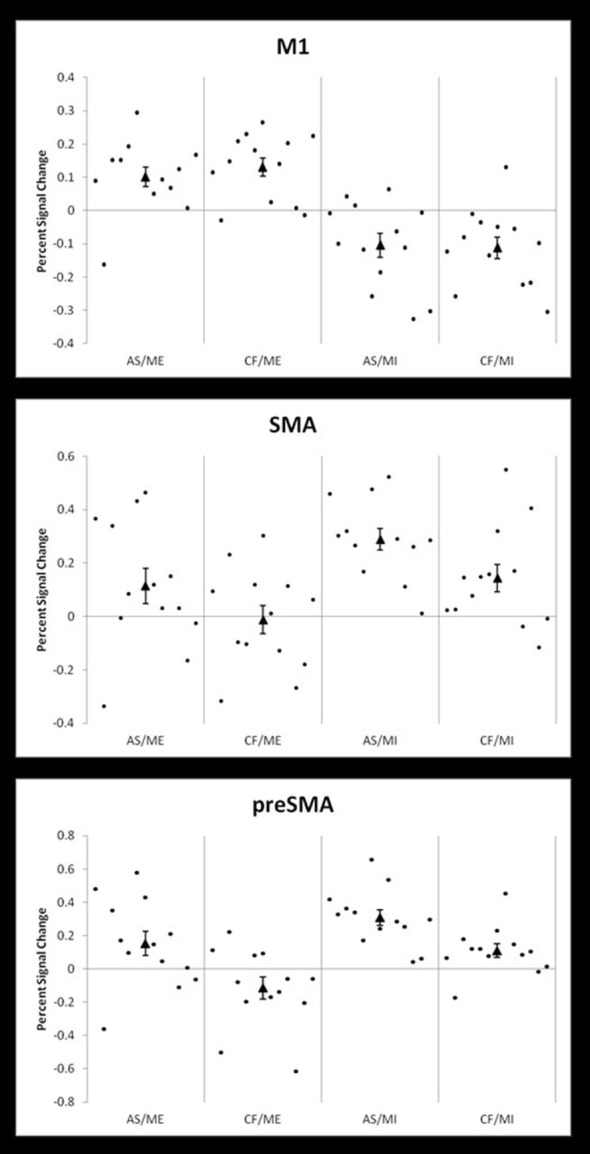
**Percent signal changes in M1, SMA and pre-SMA across conditions**. Individual participant percent signal changes across the four conditions; action selection/motor execution (AS/ME), command following/motor execution (CF/ME), action selection/motor imagery (AS/MI), and command following/motor imagery (CF/MI). Results are shown in three 10 mm, spherical ROIs defined by coordinates derived from group level activity from the whole brain analysis. The top panel shows activity in the primary motor cortex (M1, coordinates −30 −25 58, from the motor execution vs. rest contrast), which also includes primary somatosensory cortex. The middle panel shows activity in the supplementary motor area (SMA; coordinates −3 8 55, from the motor imagery vs. rest contrast) and the bottom panel shows activity in the pre-SMA (preSMA, coordinates 3 20 46, from the positive effect of level of selection contrast). The group mean percent signal change is indicated by a triangle.

Additionally, we calculated the percent signal change for each participant in three 10 mm spherical ROIs (i.e., M1, SMA, and pre-SMA). These were defined using coordinates from the clusters revealed in the group level whole brain analysis for ME vs. rest (coordinates −30 −25 58), MI vs. rest (−3 8 55), and the positive effect of level of selection (3 20 46). Figure 4 shows the percent signal change across all four conditions for each ROI: AS/ME, CF/ME, AS/MI, and CF/MI. Activity in M1 increased during ME conditions compared to imagery, while activity in the SMA followed the opposite pattern, consistently across participants. The pre-SMA appeared to become more activated in conditions where participants select their own actions compared to following commands. Activity in M1 did not differ between the AS and CF condition within a given modality (execution or imagery).

## Discussion

Here, we provide the first report of the differences in brain activity elicited by CF and the level of response selection necessary for binary communication, in an fMRI task involving both external behavioral responses (i.e., ME), as well as covert neural responses (i.e., MI). Our results provide evidence to support that, while motor imagery and execution may be dissociable processes, the mechanisms underlying the ability to select between two actions are not dependent on how the motor response is expressed (i.e., executed or imagined).

While both behavioral and neuroimaging studies agree in suggesting that most VS or MCS patients who are overtly or covertly able to follow commands do not show communication abilities (Monti et al., 2010; Owen, 2011; Naci and Owen, 2013; Estraneo et al., 2014) the neural correlates behind this divergence are not well understood. We found significantly higher activity in frontal regions, including the pre-SMA, and middle frontal gyrus when participants had to select between two possible actions (pre-requisite for binary communication), as compared to when the examiner determined each specific action (command following). Previous studies have demonstrated that both areas are involved with higher order executive functions related to voluntary motor control (Wiese et al., 2004; Haggard, 2008; Mostofsky and Simmonds, 2008). The cluster of activation in the left middle frontal gyrus extended to the dorsolateral prefrontal cortex (DLPFC). Activity in this region has been reported in several PET studies (Jahanshahi et al., 1995; Jenkins et al., 2000; Weeks et al., 2001) comparing externally-triggered movements (e.g., cued by an auditory or visual stimulus) to self-initiated movements (e.g., self-paced by the participant). DLPFC involvement was thought to reflect an increased demand on working memory in the self-initiated condition, where participants had to keep track of their own movements’ timing rather than simply responding to cues (Weeks et al., 2001). In our experiment, participants determined the type, rather than the timing, of their movements in the AS condition. Nevertheless, the greater activity elicited in the DLPFC likely also reflects working memory demands, as they had to hold their selected movements in memory to create their unique movement sequences.

Furthermore, activity in pre-SMA has previously been observed in motor experiments where participants were asked to control certain aspects of the movements they performed, such as direction, timing, or type (Jahanshahi et al., 1995; Deiber et al., 1999; Jenkins et al., 2000; Jankelowitz and Colebatch, 2002; Gowen and Miall, 2007). Crucially, the pre-SMA has also been related to decision-making and AS processes (Gleichgerrcht et al., 2010). In a 2008 review, Haggard (2008) proposed that voluntary action execution is a form of decision-making that includes two decisions: whether to act, and what to do. The latter is further broken down into selecting between a goal or task, and selecting between possible movements to achieve it, both of which involve participation of the pre-SMA. Moreover, neurophysiological studies in non-human primates have revealed increased activity in the pre-SMA when animals must select between different motor responses cued by visual stimuli (Matsuzaka et al., 1992; Isoda and Hikosaka, 2007). Similarly, neuroimaging studies in humans have shown that regions within the pre-SMA are activated when participants choose between different movements (Hoffstaedter et al., 2013), and different tasks (e.g., following a specific, cued movement plan or making their own movement plan) as well as quickly switching between these two tasks (Nachev et al., 2005). The pre-SMA is thought to help form and initiate action intentions by forwarding inputs from the basal ganglia and prefrontal cortex to the SMA and M1 (Nachev et al., 2007; Haggard, 2008). Prefrontal areas including the pre-SMA are also thought to influence AS by preferentially enhancing a particular desired action among several alternatives represented in the parietal cortex (Cisek and Kalaska, 2010). Our results also showed pre-SMA involvement when participants selected actions that were imagined. Indeed, neuroimaging studies suggest that the pre-SMA and SMA are also recruited in the motor planning and preparation phase of imagined movement (Stephan et al., 1995; Cunnington et al., 2005). Additionally, we found activity in the left insula and right angular gyrus for AS, as compared to CF. These areas are known to be involved in contributing to the sense of agency or personal authorship of voluntary movements (Farrer and Frith, 2002; Farrer et al., 2003, 2008; Tsakiris et al., 2007). Finally, we also observed a nearly significant (*p* = 0.054) cluster of activity in the left supramarginal gyrus. This is in agreement with a previous fMRI study that reported increased activation in the left inferior parietal lobe when subjects self-initiated finger movements, compared to when these movements were performed in response to visual cues (Wiese et al., 2004).

The inverse contrast comparing activity when participants followed commands to when they selected their own responses revealed activity in primary auditory cortices. The auditory activity likely reflects the increased auditory cues (i.e., movement words) presented in the CF blocks, compared to the simpler “Go” cues heard during AS. Interestingly, this contrast also revealed activity in the precuneus, an area associated with consciousness and its disorders (Cavanna and Trimble, 2006). Specifically, the precuneus is part of the default mode network (DMN), a long-range brain network thought to be active during resting-state and self-referential thought (Uddin et al., 2009). The DMN’s functional and structural connectivity seems to be associated with the level of impairment in DOC patients, with increased connectivity associated with higher levels of consciousness (Vanhaudenhuyse et al., 2010; Fernández-Espejo et al., 2012; Crone et al., 2015). Importantly, the DMN has been found to deactivate during tasks requiring effortful attention to external stimuli and goal-oriented responses (Singh and Fawcett, 2008; Uddin et al., 2009). Therefore, it is possible the precuneus activity seen in this contrast represents deactivation in this area during the more cognitively demanding AS condition.

Overall, our results suggest that selecting between two possible actions requires a greater involvement of high-level associative areas in frontal and parietal cortices than following simple commands. Crucially, recent structural and functional connectivity studies have revealed marked impairments in associative fronto-parietal networks in DOC patients (Laureys et al., 1999; Juengling et al., 2005; Laureys, 2005; Levine et al., 2008; Fernández-Espejo et al., 2012), which correlated with the complexity of the behaviors the patients were able to exhibit (Fernández-Espejo et al., 2012). Although patients who are capable of following commands vs. those who can also communicate have not been specifically compared in the studies above, a reasonable hypothesis would be that more severe disruptions in these long-range fronto-parietal networks may be the basis for the inability to communicate that some “command followers” present. Future studies directly comparing brain damage in these two groups of patients are needed to test this hypothesis, and identify the specific structural damage that underlies this dissociation.

A central aim of our study was to investigate whether the differences between CF and AS for binary communication were dependent on the modality in which the participant expressed their response (i.e., imagery or execution). MI involves creating an internal mental representation of an overt action without any concurrent executed movement (Jeannerod, 1995). In contrast, ME involves physically performing a movement. Classic neuroimaging studies revealed similar patterns of brain activity for both MI and ME (Porro et al., 1996; Lotze and Halsband, 2006). This led some authors to conclude they may be equivalent processes (Jeannerod, 1995; Stephan et al., 1995). However, more recent work has revealed important differences in functional brain activation and connectivity between the two (Kilner et al., 2004; Carrillo-de-la-Peña et al., 2008; Burianová et al., 2013; Machado et al., 2013; Xu et al., 2014; Fernández-Espejo et al., in press). Consistent with these reports, we identified higher activation in left sensorimotor areas, including M1, S1, and the superior parietal lobule, for ME, as compared to MI. In contrast, MI was associated with higher activity in the right M1 and S1. This higher ipsilateral activity may be reflecting inhibition during ME. Indeed, concurrent left M1 activation and right M1 deactivation has been previously reported in both EEG and fMRI studies involving right hand movement (Grefkes et al., 2008; Hayashi et al., 2008; Machado et al., 2013).

A large number of studies have reported covert CF and/or communication in patients who are entirely non-behaviorally responsive (Cruse et al., 2011, 2012a; Goldfine et al., 2012; Owen et al., 2006; Schnakers et al., 2008; Monti et al., 2010, 2014; Bardin et al., 2011; Bekinschtein et al., 2011; Fernández-Espejo and Owen, 2013; Lulé et al., 2013; Naci and Owen, 2013; Forgacs et al., 2014; Gibson et al., 2014; Pan et al., 2014; Coyle et al., 2015). However, some reports suggest that the opposite discrepancy between overt and covert capabilities may also exist. For instance, Bardin et al. (2011) reported two brain injured patients, from a cohort of seven, who were capable of following commands or communicating in behavioral assessments, but failed to do so with MI paradigms. The authors suggested that resource allocation problems relative to the high cognitive demands of MI tasks could account for their results. An alternative explanation, however, would be that the above discrepancies simply represent false-negatives in the neuroimaging data. While the prevalence of false negative results in VS patients is difficult to estimate because of the lack of a reliable “gold-standard” clinical measure to confirm whether a patient is conscious or not (Peterson et al., 2013), it is well known that a small proportion (15%) of conscious, healthy volunteers fail to show reliable appropriate brain activity in MI paradigms (Cruse et al., 2011; Hampshire et al., 2012; Fernández-Espejo et al., 2014). Furthermore, abnormal or absent brain activity in these patients could result from various factors including their unique brain damage and arousal levels, as well as limitations with the neuroimaging technique used (e.g., excessive motion artifacts).

Crucially, here we failed to identify an interaction between the response modality (i.e., MI and ME) and the level of AS. Although our relatively small sample size (*n* = 13) raises the possibility that this null result could be a false negative (Peterson et al., 2015), the observed lack of interaction persisted even at very low statistical thresholds. When a mask including all areas active in main effects was used at a single subject level, the positive interaction yielded activity in only two participants, which did not survive peak or cluster level correction. This lack of evidence for an interaction suggests that these two factors are dissociable. A more in-depth analysis of each individual’s percentage of signal change in M1, SMA and pre-SMA across conditions supported this claim. In other words, our results may suggest that the neural processes involved in following a command or selecting an action are not dependent on the modality in which the response is expressed. Therefore, we provide evidence to suggest that a patient who can communicate by selecting between two mental responses in an fMRI paradigm would be demonstrating the same level of cognitive function as a patient communicating with their behavioral responses at the bedside. This provides further support for the use of MI fMRI tasks as a reliable proxy for overt CF and communication in brain-injured patients.

## Author Contributions

NRO, AMO and DF-E designed and conceived of this fMRI study. NRO and DF-E collected and analyzed the fMRI data and drafted the manuscript, and AMO provided critical feedback on the manuscript. All authors read and approved the final manuscript.

## Conflict of Interest Statement

The authors declare that the research was conducted in the absence of any commercial or financial relationships that could be construed as a potential conflict of interest.

## Footnotes

^1^http://www.fmrib.ox.ac.uk./fsl

^2^http://www.fil.ion.ucl.ac.uk/spm
